# Serum HBV DNA plus RNA reflecting cccDNA level before and during NAs treatment in HBeAg positive CHB patients

**DOI:** 10.7150/ijms.71737

**Published:** 2022-05-09

**Authors:** Yang Wang, Yanna Liu, Hao Liao, Zhongping Deng, Dandan Bian, Yan Ren, Guangxin Yu, Yingying Jiang, Li Bai, Shuang Liu, Mei Liu, Li Zhou, Yu Chen, Xinyue Chen, Zhongping Duan, Fengmin Lu, Sujun Zheng

**Affiliations:** 1 Liver disease center, Beijing YouAn Hospital, Capital Medical University, Beijing 100069, China.; 2 Beijing Municipal Key Laboratory of Liver Failure and Artificial Liver Treatment & Research, Beijing YouAn Hospital, Capital Medical University, Beijing 100069, China.; 3 Department of Microbiology and Infectious Disease Center, School of Basic Medical Sciences, Peking University Health Science Center, Beijing 100191, China.; 4 Department of Clinical Laboratory, Shenzhen Third People's Hospital, Southern University of Science and Technology, National Clinical Research Center for Infectious Diseases, Shenzhen, 518112, PR China.; 5 Academy for Advanced Interdisciplinary Studies, Peking University, Beijing 100871, China.; 6 Hunan Provincial Key Laboratory of Gene Diagnostic Technology, Changsha 410205, China.

**Keywords:** chronic hepatitis B, pregenomic RNA, covalently closed circular DNA, nucleos(t)ide analogues, hepatitis B surface antigen

## Abstract

**Background & Aims:** Correlations between serum viral markers and intrahepatic cccDNA in patients undergoing long-term nucleos(t)ide analogues (NAs) treatment haven't been fully explored. In this study, we evaluate the correlation between intrahepatic cccDNA and other serum viral markers and intrahepatic HBV DNA in HBeAg positive chronic hepatitis B (CHB) patients during 60-month treatment with NAs.

**Methods:** Fifty-four HBeAg positive CHB patients received long-term NAs treatment were included in this study. Serial serum samples were regularly collected and quantitatively analyzed for HBsAg, HBV DNA, HBV RNA and HBcrAg. Histological samples from liver biopsy at baseline and month 60 were analyzed for intrahepatic HBV DNA and cccDNA.

**Results:** At baseline, serum HBV DNA plus RNA was positively associated with intrahepatic cccDNA in multivariate regression analysis (β=0.205, *P*<0.001). In the correlation analysis between cccDNA and serum viral markers, HBV DNA plus RNA had the highest correlation coefficient (r=0.698, *P*<0.001), followed by serum HBV DNA (r=0.641, *P*<0.001), HBV RNA (r=0.590, *P*<0.001), and HBcrAg (r=0.564, *P*<0.001). At month 60, correlations between these serum viral markers and cccDNA were not observed (*P*>0.05). Multivariate regression analysis showed that only the decreased HBV DNA plus RNA was positively associated with cccDNA decline (β=0.172, *P* =0.006). Changes of HBV DNA plus RNA (r=0.525, *P*=0.001) was better correlated with cccDNA decline as compared to HBV RNA (r=0.384, *P*=0.008), HBV DNA (r=0.431, *P*=0.003), and HBsAg (r=0.342, *P*=0.029).

**Conclusions:** Serum HBV DNA plus RNA better correlated with intrahepatic cccDNA than other viral makers before and during NAs treatment in HBeAg positive CHB patients.

## Introduction

Hepatitis B virus (HBV) infection has been a global public health challenge 1. Nucleos(t)ide analogues (NAs) suppress HBV DNA synthesis via inhibiting reverse transcription of pregenomic RNA (pgRNA) into HBV DNA, which can reverse liver fibrosis and reduce the risk of hepatocellular carcinoma 2. Although NAs do not directly affect the covalently closed circular DNA (cccDNA), NAs could efficiently inhibit the replenishment of cccDNA pool via blocking relaxed circular DNA (rcDNA) formation 3-5. Clinical studies also observed significant decrease of intrahepatic cccDNA after long-term NAs treatment in CHB patients 6,7, and intrahepatic cccDNA level decline is the most direct prognostic indicator of response to antiviral treatment 8. However, the invasive procedure and potential sampling error restricted the utilization of intrahepatic cccDNA in clinical practice. Therefore, exploring noninvasive and convenient serum viral markers that indirectly reflecting intrahepatic cccDNA level has important clinical value in CHB patient receiving long-term NAs treatment.

Classical indicators, HBsAg and HBV DNA, have been thought to be positively correlated with intrahepatic cccDNA before NAs treatment, but this correlation is weak 9-11. Besides, HBV DNA can be efficiently inhibited to an undetectable level in a majority of CHB patients after NAs treatment, and the integrated HBV DNA fragments could also generate HBsAg 12, all these factors may influence the relationship between serum HBsAg, HBV DNA and intrahepatic cccDNA level before and after NAs treatment 13,14.

Novel serum viral markers, HBV RNA 3, 14-16 and HBV core-related antigen (HBcrAg) 17-19 have been proposed as indicators to reflect intrahepatic transcriptional activity of cccDNA in CHB patients. Serum HBV RNA levels were positively correlated with intrahepatic cccDNA before NAs treatment but the correlation disappeared after 96 weeks of NAs treatment in HBeAg positive CHB patients 14. Besides, higher serum HBV RNA/DNA ratio indicated the lower reverse transcription of pgRNA 20,21. Huang et al. reported that serum HBV DNA plus RNA exhibited superiority than HBV RNA or HBV DNA alone in reflecting cccDNA activity in treatment-naive HBeAg positive CHB patients 21. Recent researches have reported serum HBcrAg had a better correlation with intrahepatic cccDNA level before antiviral treatment 18, 22. So far, it is still lacking universally acknowledged indicator to reflect cccDNA level. Whether these serum viral markers are consistently correlated with cccDNA after long-term NAs treatment remains unknown. In addition, whether the changes of these serum viral markers reflected the decline of cccDNA during NAs treatment remains to be elucidated. These studies intrigue us to conduct a head-to-head comparison of serum HBsAg, HBV RNA, HBV DNA, HBV DNA plus RNA, HBV RNA/DNA and HBcrAg levels in reflecting intrahepatic cccDNA in CHB patients treated with NAs.

In this single center, longitudinal study, we evaluate the correlation between intrahepatic cccDNA and serum HBsAg, HBV RNA, HBV DNA, HBV DNA plus RNA, HBV RNA/DNA, HBcrAg, as well as intrahepatic HBV DNA in HBeAg positive CHB patients with 60-month NAs treatment.

## Methods

### Patients and study design

This study was conducted using a cohort of 83 HBeAg positive CHB patients receiving NAs monotherapy. Of them, 54 patients with liver biopsy at baseline and month 60 were included in this analysis. Patients of this cohort were prospectively recruited from Beijing YouAn Hospital, Capital Medical University (Beijing, China) between June 2007 and July 2008. Eligible patients were diagnosed CHB according to the American Association for the Study of Liver Diseases guideline 23, male or female patients aged ≥ 16 years. The exclusion criteria were as follows: i) co‑infection with another viruses, including hepatitis C or D virus, Epstein-Barr virus, cytomegalovirus and human immunodeficiency viruses, the existence of autoimmune liver disease, or alcoholic liver disease; ii) with decompensated liver function (ascites, hepatic encephalopathy or upper gastrointestinal bleeding); iii) with any diseases of other major organs, such as severe heart disease or kidney disease; iv) poor compliance; v) history of a malignancy, including hepatocellular carcinoma, carcinoma *in situ* and atypical hyperplastic nodules; vi) with mental illness; vii) had received corticosteroids, immunosuppressants or chemotherapeutic drugs ≤6 months prior to enrollment; and viii) pregnant or breast‑feeding women.

At enrollment and 60 months on-treatment, serum specimens were collected for liver function tests, viral marker tests and HBV DNA quantification. Remaining serum samples were stored at -80 °C for subsequent research. Percutaneous liver biopsy were performed to evaluate the histology, which were diagnosed according to the modified Knodell and Ishak scoring system 24.

With the foregoing collected blood, we quantified HBsAg, HBV RNA and HBcrAg levels at the time point of baseline and month 60 of NAs treatment. Intrahepatic HBV DNA and cccDNA levels at baseline and month 60 were measured by a method as previously reported 21.

The study was conducted in compliance with the Declaration of Helsinki. Use of the research samples was approved by the Medical Ethics Review Committee of Beijing YouAn Hospital. All patients provided written informed consent authorizing us to access their medical records and to store the remaining serum specimens for research purposes.

### Assays for serological HBV markers, HBV DNA, HBV RNA, and HBcrAg

Serum HBsAg, anti-HBs, HBeAg, anti-HBe, and anti-HBc were determined on a Roche Cobas e601 analyzer using an electrochemiluminescence immunoassay (Abbott Laboratories, Chicago, IL, USA). HBsAg was quantified using an Elecsys for HBsAg quantitation (Roche Diagnostics) with a lower limit of detection (LLD) of 0.05 IU/mL. The serum HBV DNA level was determined using the Cobas HBV Amplicor Monitor assay (Roche Diagnostics, Pleasanton, CA, USA), with a LLD of 50 IU/mL. Serum HBV RNA level was determined as described previously 15, 21, 25. Briefly, HBV RNA was isolated with the nucleic acid extraction or purification kit (Sansure Biotech, Changsha, China) and treated with DNase I (Thermo Fisher Scientific, Waltham, MA, USA). The specially modified super-cis nano-magnetic beads efficiently adsorbed and enriched nucleic acids from 200 µL serum. For DNase I treatment, every reaction mixture comprised 2 µL of DNase I Reaction Buffer (10×), 2 µL of DNAse I (RNase-free), and 16 µL of total nucleic acids. The reaction was carried out at 37 °C for 30 min. Next, each mixture was incubated at 75 °C for 10 min to inactivate DNase I. Finally, DNase-I-treated HBV RNA was one-step of reverse-transcribed and real-time fluorescent quantitative PCR using the HBV pgRNA high-sensitivity quantitative kit (Sansure Biotech, Changsha, China). The LLD of the assay was 200 copies/mL. Details for HBV RNA assay could be found in Supplementary Materials. HBcrAg was determined using chemiluminescent enzyme immunoassay in automated analyzer system (Lumipulse System, Fujirebio Inc., Tokyo, Japan). The LLD was 1,000 U/mL with a linear range of 3-7 log10 U/mL.

### Quantitation of intrahepatic HBV DNA and cccDNA

About 30 μm formalin fixation and paraffin embedding (FFPE) liver biopsy tissue in sections of 6 μm each was used for DNA extraction. The DNA was extracted using QIAamp FFPE DNA Mini Kit (QIAGEN, GmbH, Hilden, Germany) according to the instructions of the manufacturer. T5 Exonuclease (New England Biolabs, USA) was used to digest HBV rcDNA, replicative dsDNA and ssDNA. The reaction mixture contained 100 ng extracted DNA, 0.5 µL (10 units) T5 Exonuclease, 1 µL NEBuffer 4 (10×) with Nuclease-free H2O to a final volume of 10 μL. The digestion was carried out at 37 °C for 1 h, and stop reaction with EDTA to at least 11 mM. We combined 6.42 μL of digestion product, which was obtained in the previous step, with 7.50 μL QuantStudio™ 3D Digital PCR Master Mix, 0.06 μL of TaqMan Probe-RC-MGB (50 μM), 0.06 μL TaqMan Probe-RNAseP-VIC (50 μM), 0.24 μL primer of rc-F, 0.24 μL primer of rc-R, 0.24 μL primer of RNaseP-F and 0.24 μL primer of RNaseP-R. This sample mix 15 μL was added on each chip and loaded on ProFlex™ 2x Flat PCR System with the following program: Absolute quantification was determined using QuantStudio™ 3D Digital PCR System (Thermo Fisher Scientific Inc., Waltham, Massachusetts, USA) and analyzed with QuantStudio 3D AnalysisSuite Cloud Software. (https://china.apps.thermofisher.com/quantstudio3d/). All intrahepatic HBV cccDNA values were normalized to cell number assessed by RNase P copy number assay.

### Statistical analysis

Data were analyzed using the IBM SPSS 22.0 (SPSS Inc., Chicago, IL, USA) and R software version 4.1.2 (R Foundation for Statistical Computing, Vienna, Austria). *P*<0.05 was considered to be statistically significant in two-tailed test. HBsAg, HBV DNA, HBV RNA, HBcrAg, intrahepatic HBV DNA and cccDNA expression were logarithmically transformed for analysis. HBV DNA plus RNA was measured using the numbers of log_10_ HBV DNA plus log_10_ HBV RNA copies/mL, the ratio between HBV RNA and DNA was also assessed using the ratio of log_10_ HBV RNA copies/mL to log_10_ HBV DNA, as previously described 21, 26. Continuous variables with normal distribution were expressed as mean ± SD (standard deviation). Continuous variable with abnormal distribution were expressed as median and range. Dichotomy variables were expressed as counting or proportion. The comparison of continuous quantitative data between before and after treatment was performed using *Wilcoxon rank sum test*. Moving forward (LR) multivariate linear regression analysis was performed to determine factors associated with intrahepatic HBV cccDNA levels, and the *P* values of entry and removal were respectively set to 0.05 and 0.1. Correlation between two continuous variables was also calculated and visualized using the R packages 'GGally' and 'ggplot2'.

## Results

### Characteristics of CHB patients

A total of 54 patients with liver biopsy at baseline and month 60 were included in this study. Of them, 24 were NAs-naïve patients, 30 were previously Lamivudine exposure patients. Twenty-eight (51.85%) patients were treated with Entacavir (ETV) 0.5 mg once daily and the other 26 (48.15%) patients were treated with Adefovir dipivoxil (ADV) 10mg once daily, the differences of baseline characteristics between these two groups were not significant (all *P* > 0.05), detailed information were shown in Supplementary Table 1. Forty-seven (87.04%) patients were male with an average age of 36.54 ± 9.44 years. Twenty-eight patients with available genotype data were analyzed, with 20 (71.43%) were genotype C. Median inflammation and fibrosis score were 7 (range, 2-15) and 3 (range, 1-5), respectively.

During 60 months' treatment, one previous lamivudine exposure patient in ETV group was detected ETV-associated resistant variants rtV173M, rtL180M, rtM204V/I and rtT184F. Four patients in ADV group were detected ADV-associated resistant variants rtA181 V/T or rtN236T. The levels of HBsAg, HBV DNA, HBV RNA, HBV RNA plus RNA, HBcrAg, intrahepatic HBV DNA and cccDNA were all significantly decreased (*P* <0.001). Inflammation and fibrosis score were also significantly decreased (*P* <0.001), while the ratio of HBV RNA/DNA was significantly increased (*P* <0.001). The detailed information were shown in Table 1.

### Regression analysis of factors associated with intrahepatic cccDNA

At baseline, univariate linear regression analysis showed that serum HBV DNA, HBV RNA, HBV DNA plus RNA, HBcrAg, intrahepatic HBV DNA, and Ishak fibrosis score were all associated with cccDNA (all *P* <0.05), while HBsAg, HBV RNA/DNA and inflammation score were not associated with cccDNA (all *P* >0.05) Table 2. The multivariate regression analysis showed that only HBV DNA plus RNA was positively associated with cccDNA (β=0.205, 95%CI: 0.135-0.274, *P* <0.001).

At month 60 of NAs treatment, univariate linear regression analysis showed that intrahepatic HBV DNA and Ishak fibrosis score were associated with cccDNA (both *P*<0.05). HBsAg, HBV DNA, HBV RNA, HBV RNA/DNA, HBV DNA plus RNA, HBcrAg and inflammation score were not associated with cccDNA in our study (*P* >0.05), Table 2.

During 60 months' NAs treatment, univariate linear regression analysis showed that the decreased HBsAg, HBV DNA, HBV RNA, HBV DNA plus RNA, intrahepatic HBV DNA and inflammation score were associated with cccDNA decline (all *P*<0.05). The changed HBV RNA/DNA, HBcrAg and fibrosis score were not associated with cccDNA decline (all *P* >0.05) Table 3. Multivariate regression analysis showed that only decreased HBV DNA plus RNA was positively associated with cccDNA decline (β=0.172, 95%CI: 0.054-0.289, *P* =0.006).

### Correlation analysis between cccDNA and serum biomarkers, as well as intrahepatic HBV DNA

**Figure 1:**
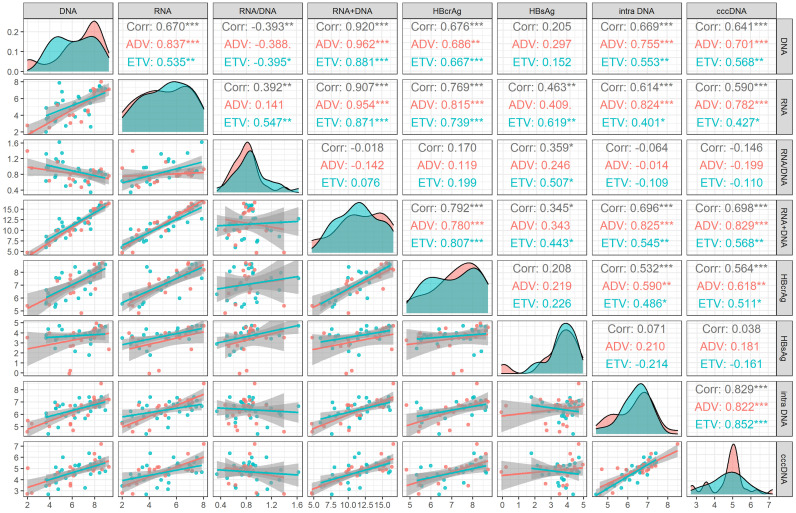
Correlation analysis of viral markers at baseline in HBeAg positive patients. ADV, Adefovir dipivoxil; cccDNA, covalently closed circular DNA; corr, correlation coefficient; ETV, Entacavir; HBsAg, hepatitis B surface antigen; HBcrAg, hepatitis B core-related antigen; intra DNA, intrahepatic HBV DNA; RNA+DNA, HBV RNA plus DNA; RNA/DNA, HBV RNA to HBV DNA ratio. *** indicated *P* < 0.001, ** indicated *P* < 0.01, * indicated *P* < 0.05.

We analyzed the correlation between viral markers and cccDNA both at baseline and month 60 of NAs treatment. At baseline, HBV DNA plus RNA had the highest correlation with cccDNA (r=0.698, *P*<0.001), followed by HBV DNA (r=0.641, *P* <0.001), HBV RNA (r=0.590, *P* <0.001) and HBcrAg (r=0.564, *P*<0.001), which was lower than the correlation coefficient between intrahepatic HBV DNA and cccDNA (r=0.829, *P* <0.001). HBsAg and HBV RNA/DNA had no correlation with cccDNA (*P* = 0.804 and 0.317, respectively). The correlation of different drug subgroups were shown in Figure 1.

Based on the median value of inflammation and fibrosis score, we further studied whether the correlations between serum viral markers and cccDNA were influenced by inflammation and fibrosis. The results showed that neither inflammation score (≤7 vs. >7) nor fibrosis score (≤3 vs >3) influence the correlations between intrahepatic cccDNA and serum HBsAg, HBV DNA, HBV RNA, HBV RNA/DNA and HBV DNA plus RNA. The correlation between intrahepatic cccDNA and HBcrAg was not influenced by inflammatory (≤7 vs >7), but the correlations was influenced by fibrosis (≤3 vs >3), as shown in Table 4.

**Figure 2:**
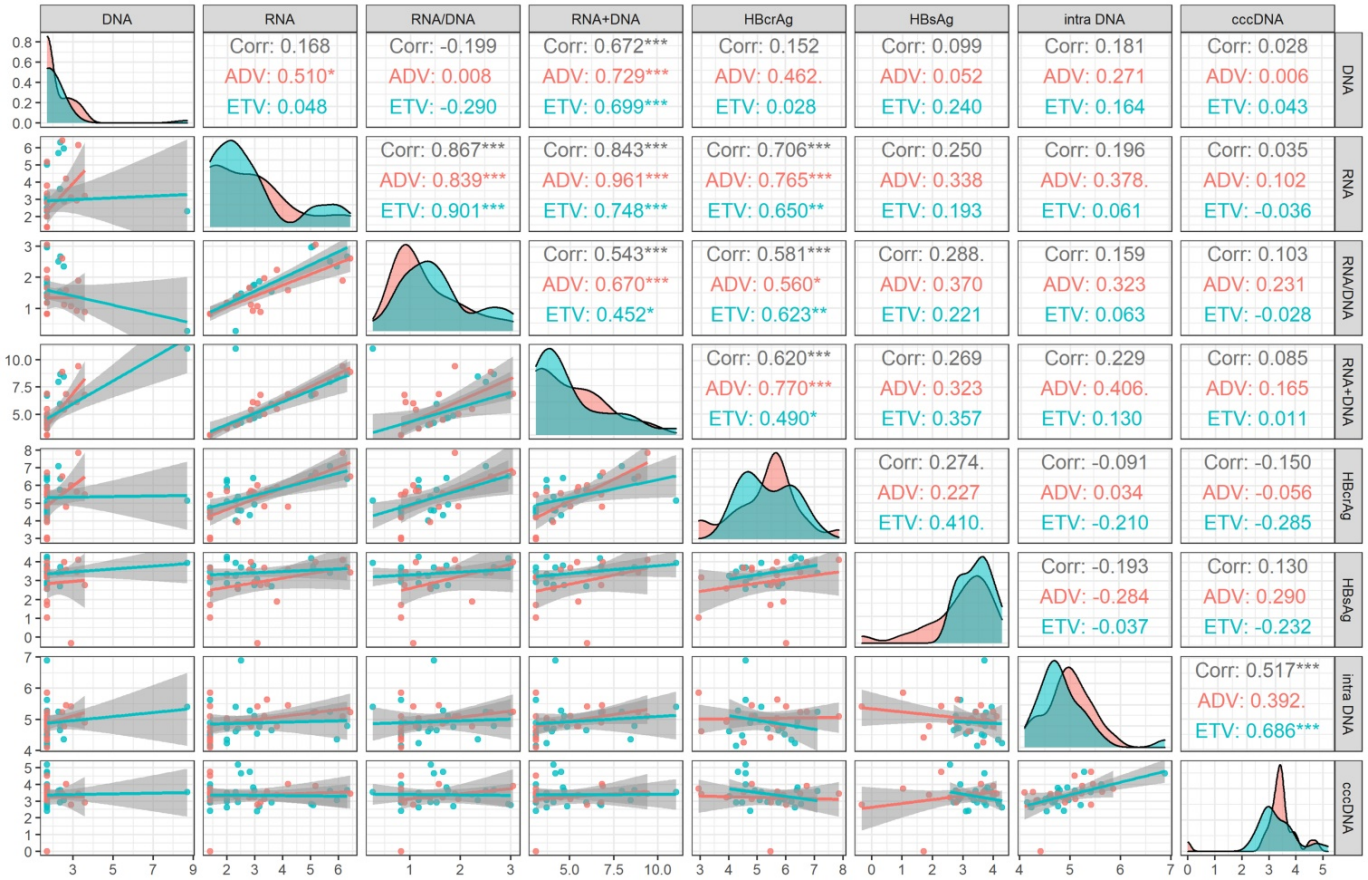
Correlation analysis of viral markers at month 60 in HBeAg positive patients. ADV, Adefovir dipivoxil; cccDNA, covalently closed circular DNA; corr, correlation coefficient; ETV, Entacavir; HBsAg, hepatitis B surface antigen; HBcrAg, hepatitis B core-related antigen; intra DNA, intrahepatic HBV DNA; RNA+DNA, HBV RNA plus DNA; RNA/DNA, HBV RNA to HBV DNA ratio. *** indicated *P* < 0.001, ** indicated *P* < 0.01, * indicated *P* < 0.05.

At month 60 of NAs treatment, intrahepatic HBV DNA was still significantly correlated with cccDNA level (r=0.517, *P*<0.001). None of the serum viral markers (HBsAg, HBV DNA, HBV RNA, HBV RNA/DNA, HBV DNA plus RNA, and HBcrAg) were correlated with cccDNA level. The correlation of different drug subgroups were shown in Figure 2.

**Figure 3:**
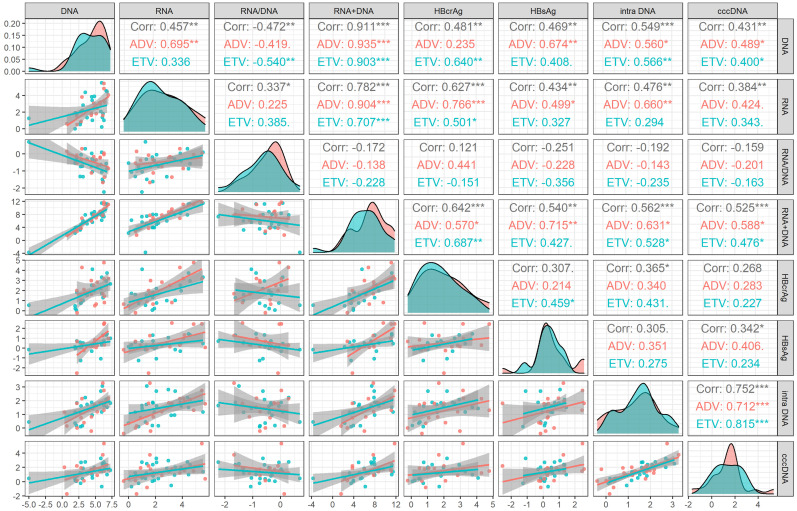
Correlation analysis of the changed value of viral markers after 60 months' treatment in HBeAg positive patients. ADV, Adefovir dipivoxil; cccDNA, covalently closed circular DNA; corr, correlation coefficient; ETV, Entacavir; HBsAg, hepatitis B surface antigen; HBcrAg, hepatitis B core-related antigen; intra DNA, intrahepatic HBV DNA; RNA+DNA, HBV RNA plus DNA; RNA/DNA, HBV RNA to HBV DNA ratio. *** indicated *P* < 0.001, ** indicated *P* < 0.01, * indicated *P* < 0.05.

During 60 months' treatment, the correlation coefficient between cccDNA decline and decreased serum HBV RNA plus DNA (r=0.525, *P*=0.001) was higher than that of HBV DNA (r=0.431, *P*=0.003), HBV RNA (r=0.384, *P*=0.008), HBsAg (r=0.342, *P*=0.029), HBcrAg (r=0.268, *P*=0.103) and HBV RNA/DNA (r=-0.159, *P*=0.334), only lower than the correlation coefficient between cccDNA decline and decreased intrahepatic HBV DNA (r=0.752, *P*<0.001), the correlation of different drug subgroups were shown in Figure 3.

### Correlation coefficients changes between HBV DNA plus RNA and other serum viral markers

The correlation coefficients between HBV DNA plus RNA and HBsAg (r=0.269, *P*=0.112), HBV DNA (r=0.672, *P*<0.001) HBV RNA (r=0.843, *P*<0.001) and HBcrAg (r=0.620, *P*<0.001) at month 60 were lower than these corresponding correlation coefficients at baseline [HBsAg (r=0.345, *P*=0.025), HBV DNA (r=0.920, *P*<0.001), HBV RNA(r=0.907, *P*<0.001), and HBcrAg (r=0.792, *P*<0.001)]. On the contrary, the correlation coefficient between HBV DNA plus RNA and HBV RNA/DNA at month 60 (r=0.543, *P*<0.001) was higher than that at baseline (r=-0.018, *P* =0.905), as shown in Figure 1 and Figure 2.

## Discussion

Based on real-life clinical practice, the present study represented a head-to-head comparison of these serum viral markers in reflecting intrahepatic cccDNA levels. We found that baseline serum HBV DNA plus RNA was better positively associated with cccDNA than HBsAg, HBV RNA, HBV DNA, HBV RNA/DNA and HBcrAg in HBeAg positive CHB patients, and this correlation were stable in patients with different inflammation and fibrosis scores. However, this association disappeared after 60 months' NAs treatment. Furthermore, the decrease of HBV DNA plus RNA was also better positively correlated with the decline of intrahepatic cccDNA than other serum viral markers during 60 months NA treatment.

According to our results, serum viral markers positively correlated with cccDNA in HBeAg positive CHB patients at baseline, but this correlation was not consistent after 60 months' NAs treatment. This was consistent with previous studies that serum HBV RNA was positively correlated with cccDNA level before NAs treatment in HBeAg positive CHB patients 14, 22, but was not correlated with cccDNA after 96 weeks of NAs treatment 14. However, in the same study, intrahepatic cccDNA positively correlated with HBV DNA, but not with HBsAg before NAs treatment. Interestingly, after 96 weeks' NAs treatment, intrahepatic cccDNA correlated with HBsAg, but not with HBV DNA 14. In the present study, we found that the positive correlation between cccDNA and HBV RNA plus DNA and HBcrAg lost after 60 months' NAs treatment. Consistent with previous studies 9,14, our results indicated that HBV DNA was positively correlated with intrahepatic cccDNA before NAs treatment while this correlation disappeared after NAs treatment. Besides, HBsAg was not correlated with intrahepatic cccDNA levels neither before nor after NAs treatment, the discrepancies among these studies might due to the heterogeneity of the study population.

To our knowledge, the present study was the first head-to-head comparison study of serum HBcrAg and HBV RNA plus DNA in reflecting intrahepatic cccDNA level. Previously studies have reported that serum HBcrAg may be better than HBV RNA in reflecting intrahepatic cccDNA level before treatment 22. We did found that HBcrAg positively correlated with intrahepatic cccDNA level before NAs treatment in HBeAg positive CHB patients (r=0.564, *P*<0.001). However, the correlation was weaker than that between HBV RNA and cccDNA (r=0.590, *P*<0.001), as well as between HBV RNA plus DNA and cccDNA (r=0.698, *P*<0.001). Besides, HBcrAg did not show statistical significance in the multivariate linear regression analysis.

Among the studied serum viral markers (HBsAg, HBV DNA, HBV RNA and HBcrAg), HBV DNA plus RNA achieved the highest correlation with cccDNA. This may be explained by that HBsAg is produced both from cccDNA and HBV DNA integrated into the host genome 27, negatively affected the correlation between HBsAg and cccDNA. Further, serum HBV DNA or HBV RNA alone may be insufficient to reflect the intrahepatic cccDNA since reverse transcription of pgRNA could be blocked by NAs treatment. Moreover, anti-HBe antibodies are cross-reactive with HBcAg due to the amino acid sequence homology 28, interfering against the accurate measurement of HBcrAg. All these factors may potentially influence the correlation between these serum viral markers and cccDNA. Nevertheless, further research is needed to clarify the underlying mechanism.

The promising surrogate marker should also reflect the change of cccDNA. In a previous study, compared with the decrease of serum HBV RNA (r=0.28, *P* <0.05) and HBV DNA (r =0.35, *P*=0.01) levels, the decrease of serum HBsAg levels (r =0.38, *P* <0.01) better reflected the decrease of intrahepatic cccDNA levels after the 96 weeks' NAs treatment 14. Wang *et al.* 29 have reported that the decrease of HBcrAg (r=0.282, *P*=0.043) correlated with the decline of cccDNA level after 96 weeks' NAs therapy in HBeAg positive patients. Our study found that the decrease of serum HBV DNA plus RNA (r=0.525, *P*=0.001) did the best in reflecting the decline of cccDNA among other markers, including HBV RNA (r=0.384, *P*=0.008), HBV DNA (r=0.431, *P*=0.003), and HBsAg (r=0.342, *P*=0.029) after 60 months' NAs treatment. While the decrease of HBcrAg was not correlated with the decline of cccDNA in our study, which was and inconsistent with previous study 29, the possible reason may be due to different NAs treatment course.

Our study has some limitations. The single-center design and limited sample size may bring bias to the study. Besides, considering the genotype of most patients was B or C, the results of this study should be carefully extrapolated for genotype A and D and for non-Chinese ethnicity. Future studies with a large sample size are needed to confirm the results of this study.

In conclusion, serum HBV DNA plus RNA was better than serum HBsAg, HBV RNA, HBV DNA, HBV RNA/DNA and HBcrAg in reflecting the intrahepatic cccDNA level at baseline and during 60 months' NAs treatment. This result deepens our knowledge and understanding of clinical significance of HBV RNA plus DNA in HBeAg positive CHB patients receiving long-term NAs treatment.

## Supplementary Material

Supplementary method and table.

## Figures and Tables

**Table 1:** Characteristics of HBeAg positive CHB patients

Clinical characteristics	baseline	Month 60	*P* value
Sex, male/female	47/7	-	-
Age, years	36.54±9.44	-	-
Naïve/LAM treated	24/30	-	-
Therapy drug, entecavir/adefovir	28/26	-	-
BMI Kg/m^2^	23.97±3.48	-	-
ALT, IU/L	68.30 (12.60-681.90)	22.30 (3.80-210.00)	**<0.001**
AST, IU/L	43.00 (10.90-358.80)	22.20 (11.60-73.50)	**<0.001**
AST/ALT ratio	0.65 (0.25-2.32)	1.02 (0.35-5.00)	**<0.001**
TBiL, µmol/L	15.25 (6.80-27.80)	14.10 (7.20-37.50)	0.090
ALP, U/L	89.70 (45.00-412.20)	65.60 (40.60-664.60)	**<0.001**
HBV Genotype (C/B+others)†	20/8	-	-
HBsAg (log_10_ IU/mL)	3.77 (-0.07-4.95)	3.35 (-0.33- 4.28)	**<0.001**
HBV DNA (log_10_ IU/mL)	6.60 (1.99-9.28)	1.70 (1.70-8.70)	**<0.001**
HBV RNA (log_10_ copies/mL)	5.38 (2.30-8.01)	2.40 (1.40-6.42)	**<0.001**
HBV RNA/DNA ratio	0.84 (0.34-1.62)	1.18 (0.27-3.05)	**<0.001**
HBV DNA plus RNA	11.66 (4.69-16.58)	4.21 (3.10-11.03)	**<0.001**
HBcrAg (log10 U/mL )	7.40 (4.84-8.73)	5.48 (2.95-7.86)	**<0.001**
Intrahepatic HBV DNA (log10copies/10^5^ cell)	6.60 (4.42-8.50)	4.91 (4.10-6.88)	**<0.001**
Intrahepatic cccDNA (log10copies/10^5^ cell)	4.90 (2.69-7.18)	3.34 (0.00-5.18)	**<0.001**
Hepatic inflammation grade‡	7 (2-15)	3 (1-12)	**<0.001**
Hepatic fibrosis stage‡	3 (1-5)	2(1-5)	**<0.001**

†Twenty-eight patients with available genotype data were analyzed. ‡Hepatic inflammation grade and fibrosis stage were diagnosed according to the modified knodell and Ishak scoring system respectively. Abbreviations: ADV, Adefovir; ALP, alkaline phosphatase; ALT, alanine aminotransferase; AST, aspartate aminotransferase; BMI, body mass index; cccDNA, covalently closed circular DNA; ETV, Entecavir; HBV, hepatitis B virus; HBV RNA, hepatitis B virus ribonucleic acid; HBcrAg, hepatitis B core-related antigen; HBeAg, hepatitis B e antigen; HBsAg, hepatitis B surface antigen; LAM, Lamivudine; TBIL, total bilirubin.

**Table 2:** Linear regression analysis of factors associated with intrahepatic cccDNA before and after 60 months treatment

Variables	Baseline			Month 60		
	B	95%CI	*P* value	B	95%CI	*P* value
Age, yrs	-0.003	-0.030-0.024	0.821	0.015	-0.005-0.035	0.141
Male	-0.282	-1.042-0.477	0.459	-0.265	-0.822-0.293	0.345
ETV	0.114	-0.399-0.626	0.658	0.053	-0.335-0.442	0.784
Naïve	-0.332	-0.849- 0.175	0.195	0.015	-0.375- 0.404	0.940
BMI Kg/m^2^	-0.031	-0.105-0.043	0.404	-0.016	-0.071-0.038	0.554
**HBV Genotype †**				-0.142	-0.528-0.245	0.455
B/C/others	-0.384	-0.891-0.122	0.131			
ALT, U/L	<0.001	-0.002-0.002	0.787	-0.003	-0.009-0.003	0.359
AST, U/L	<0.001	-0.004-0.004	0.988	-0.014	-0.035-0.007	0.181
AST/ALT ratio	-0.592	-1.314-0.129	0.106	0.071	-0.237-0.378	0.646
TBIL, μmol/L	<0.001	-0.048-0.049	0.990	-0.005	-0.041-0.030	0.763
ALP, U/L	-0.002	-0.007-0.002	0.308	0.005	0.001-0.011	0.067
HBsAg (log_10_ IU/mL)	0.033	-0.234-0.300	0.804	0.069	-0.179-0.317	0.576
HBV DNA (log_10_ IU/mL)	0.315	0.210-0.420	**<0.001**	0.012	-0.177-0.202	0.895
HBV RNA (log_10_ copies/mL)	0.327	0.194-0.460	**<0.001**	0.005	-0.128-0.138	0.938
HBV RNA/DNA ratio	-0.554	-1.657-0.549	0.317	0.084	-0.225-0.392	0.588
HBV DNA plus RNA	0.204	0.143-0.266	**<0.001**	0.022	-0.083-0.126	0.680
HBcrAg (log_10_ U/mL)	0.477	0.254-0.700	**<0.001**	-0.110	-0.325-0.105	0.306
Intrahepatic HBV DNA (log_10_copies/10^5^ cell)	0.926	0.752-1.100	**<0.001**	0.709	0.397-1.022	**<0.001**
Inflammation scores‡	-0.033	-0.100-0.034	0.329	0.079	-0.077-0.235	0.311
Fibrosis scores‡	-0.328	-0.575-0.082	**0.010**	0.305	0.100-0.511	**0.004**

†Twenty-eight patients with available genotype data were analyzed. ‡Hepatic inflammation grade and fibrosis stage were diagnosed according to the modified knodell and Ishak scoring system respectively. Abbreviations: ALP, alkaline phosphatase; ALT, alanine aminotransferase; AST, aspartate aminotransferase; BMI, body mass index; cccDNA, covalently closed circular DNA; ETV, Entecavir; HBV, hepatitis B virus; HBV RNA, hepatitis B virus ribonucleic acid; HBcrAg, hepatitis B core-related antigen; HBeAg, hepatitis B e antigen; HBsAg, hepatitis B surface antigen; LAM, Lamivudine; TBIL, total bilirubin.

**Table 3:** Linear regression analysis of decreased viral markers associated with intrahepatic cccDNA decline after 60 months treatment

Variables	B	95%CI	*P* value
HBsAg (log_10_ IU/mL)	0.437	0.048-0.826	**0.029**
HBV DNA (log_10_ IU/mL)	0.226	0.079-0.373	**0.003**
HBV RNA (log_10_ copies/mL)	0.314	0.085-0.543	**0.008**
HBV RNA/DNA ratio	-0.319	-0.979-0.341	0.334
HBV DNA plus RNA	0.204	0.094-0.314	**0.001**
HBcrAg (log_10_ U/mL)	0.260	-0.056-0.576	0.103
Intrahepatic HBV DNA (log_10_copies/10^5^ cell)	1.043	0.775-1.311	**<0.001**
Inflammation scores‡	-0.016	-0.212- -0.021	**0.018**
fibrosis scores‡	-0.284	-0.739-0.179	0.215

‡Hepatic inflammation grade and fibrosis stage were diagnosed according to the modified knodell and Ishak scoring system respectively. Abbreviations: cccDNA, covalently closed circular DNA; HBV, hepatitis B virus; HBV RNA, hepatitis B virus ribonucleic acid; HBcrAg, hepatitis B core-related antigen; HBeAg, hepatitis B e antigen; HBsAg, hepatitis B surface antigen.

**Table 4:** Correlation of intrahepatic cccDNA with serum viral markers stratified by inflammation and fibrosis scores

Parameters	cccDNA				cccDNA			
	Inflammation≤7		Inflammation>7		Fibrosis≤3		Fibrosis>3	
	r	P	r	P	r	P	r	P
HBsAg (log_10_ IU/mL)	0.368	0.084	-0.248	0.266	0.257	0.149	-0.420	0.175
HBV DNA (log_10_ IU/mL)	0.656	<0.001	0.632	0.001	0.526	0.001	0.880	<0.001
HBV RNA (log_10_ copies/mL)	0.580	0.003	0.596	0.002	0.496	0.002	0.688	0.007
HBV RNA/DNA	-0.401	0.052	0.068	0.747	-0.017	0.924	-0.519	0.057
HBV DNA plus RNA	0.728	<0.001	0.657	<0.001	0.611	<0.001	0.824	<0.001
HBcrAg (log_10_ U/mL)	0.567	0.006	0.519	0.019	0.592	<0.001	0.436	0.180

Hepatic inflammation grade and fibrosis stage were diagnosed according to the modified knodell and Ishak scoring system respectively. Abbreviations: cccDNA, covalently closed circular DNA; HBV, hepatitis B virus; HBV RNA, hepatitis B virus ribonucleic acid; HBcrAg, hepatitis B core-related antigen; HBeAg, hepatitis B e antigen; HBsAg, hepatitis B surface antigen.

## Abbreviations

ADV: Adefovir dipivoxil
ALT: alanine aminotransferase
AST: aspartate aminotransferase
BMI: body mass index
cccDNA: covalently closed circular DNA
CHB: chronic hepatitis B
HBV: hepatitis B virus
HBcrAg: hepatitis B core-related antigen
HBeAg: hepatitis B e antigen
HBsAg: hepatitis B surface antigen
HCC: hepatocellular carcinoma
LLD: lower limit of detection
NAs: nucleos(t)ide analogues
pgRNA: pregenomic RNA
rcDNA: relaxed circular DNA
