# Supplementary material for: Serum HBV DNA plus RNA reflecting cccDNA level before and during NAs treatment in HBeAg positive CHB patients
Source: Int J Med Sci. 2022 May 9;19(5):858–66. doi: 10.7150/ijms.71737 (PMC9149645; doi:10.7150/ijms.71737)
Supplement: Supplementary file 1 — Supplementary method and table. [file ijmsv19p0858s1.pdf]

HBV RNA Assay

HBV RNA was isolated with the nucleic acid extraction or purification kit (Sansure Biotech, Changsha, China) and treated with DNase I (Thermo Fisher Scientific, Waltham, MA, USA). The specially modified super-cis nano-magnetic beads efficiently adsorbed and enriched nucleic acids from 200 μL serum. For DNase I treatment, every reaction mixture comprised 2 μL of DNase I Reaction Buffer (10×), 2 μL of DNAse I (RNase-free), and 16 μL of total nucleic acids. The reaction was carried out at 37°C for 30 min. Next, each mixture was incubated at 75°C for 10 min to inactivate DNase I. Finally, DNase-I-treated HBV RNA was one-step of reverse-transcribed and real-time fluorescent quantitative PCR using the HBV pgRNA high-sensitivity quantitative kit (Sansure Biotech, Changsha, China). Serum HBV RNA levels were measured using SLAN-96P Real-Time PCR Systems (Shanghai Hongshi Medical Technology Co., Ltd, Shanghai, China), with the following amplification profile: an initial denaturation cycle of 1min at 95 °C, an reverse-transcription cycle of 30min at 60 °C, 95 °C for 1 min denaturation, followed by 45 cycles of denaturation for 30 s at 94 °C, annealing for 60 s at 62 °C (the fluorescence signal was collected). The amplification results were automatically analyzed. The LOD of the assay was 200 copies/mL.

Supplementary Table 1 Characteristics of HBeAg positive CHB patients

Clinical characteristics	At baseline			Changes after 60 months		
	ETV	ADV	*P* value	ETV	ADV	*P* value
Sex, male/female	25/3	22/4	0.699	-	-	-
Age, years	36.04±9.77	37.08±9.23	0.690	-	-	-
Naïve/LAM treated	14/14	10/16	0.394	-	-	-
BMI Kg/m^2	23.78±3.08	24.18±3.92	0.672	-	-	-
ALT, IU/L	58.85(12.60-337.20)	70.65(14.9-681.9)	0.287	36.60(-12.4-313.70)	47.80(-8.50-664.70)	0.451
AST, IU/L	40.50(12.90-208.60)	43.10(10.90-358.80)	0.505	18.90(-8.10-185.00)	18.65(-8.60-338.20)	0.674
AST/ALT ratio	0.80(0.29-2.32)	0.61(0.25-1.26)	0.226	-0.28(-1.03-1.25)	-0.19(-4.23-0.20)	0.985
TBiL, μmol/L	15.55±5.05	16.73±5.61	0.421	1.02(-17.6-12.00)	0.80(-21.7-14.80)	0.678
ALP, U/L	95.20(57.70-412.20)	84.60(45.00-171.00)	0.113	30.65(3.10-326.70)	14.10(-584.00-67.90)	**0.012**
HBV Genotype (C/B+others)†	8/5	12/3	0.410		-	-
HBsAg(log_{10} IU/mL)	3.78(1.78-4.72)	3.74(-0.07-4.95)	0.767	0.38(-1.26-1.45)	0.30(-2.55-2.56)	0.734
HBV DNA (log_{10} IU/mL)	6.25(3.65-8.96)	7.41(1.99-9.28)	0.350	4.45(-5.00-7.26)	4.91 (0.33-6.96)	0.589
HBV RNA (log_{10} copies/mL)	5.28±1.67	5.12±1.79	0.753	2.36±1.47	2.59±1.67	0.612
HBV RNA/DNA ratio	0.88±0.27	0.80±0.20	0.270	-0.63±0.67	-0.47±0.54	0.417

HBV DNA plus RNA	11.50±2.95	11.74±3.60	0.803	6.14±3.31	7.19±2.96	0.305
HBcrAg (log10 U/mL)	7.11±1.12	7.23±1.13	0.729	1.72±1.15	1.94±1.34	0.582
Intrahepatic HBV DNA(log10copies/10^5 cell)	6.38±0.73	6.36±0.95	0.955	1.50±0.83	1.32±0.95	0.469
Intrahepatic cccDNA(log10copies/10^5 cell)	4.71(2.69-6.35)	4.80(2.72-7.18)	0.691	1.30(-0.67-3.25)	1.55(-1.70-5.31)	0.865
Hepatic inflammation grade‡	6.5 (2-15)	7.5(3-15)	0.595	5.71±3.95	5.27±3.05	0.647
Hepatic fibrosis stage‡	3 (1-5)	3 (1-5)	0.363	1 (0-3)	1 (0-2)	0.559

†Twenty-eight patients with available genotype data were analyzed.

‡Hepatic inflammation grade and fibrosis stage were diagnosed according to the modified knodell and Ishak scoring system respectively.

Abbrevation: ADV, Adefovir; ALP, alkaline phosphatase; ALT, alanine aminotransferase; AST, aspartate aminotransferase; BMI, body mass index; cccDNA, covalently closed circular DNA; ETV, Entecavir; HBV, hepatitis B virus; HBV RNA, hepatitis B virus ribonucleic acid; HBcrAg, hepatitis B core-related antigen; HBeAg, hepatitis B e antigen; HBsAg, hepatitis B surface antigen; LAM,
Lamivudine; TBIL, total bilirubin.
